# Pancake Bonding in the Stabilization of Cationic Acene Dimers

**DOI:** 10.1021/acsmaterialsau.4c00153

**Published:** 2025-01-30

**Authors:** Rameswar Bhattacharjee, Hans Lischka, Miklos Kertesz

**Affiliations:** 1 Chemistry Department and Institute of Soft Matter, Georgetown University, 37th and O Streets, NW, Washington, DC 20057-1227, United States; 2 Department of Chemistry and Biochemistry, Texas Tech University, Lubbock, Texas 79409, United States

**Keywords:** polyaromatic hydrocarbons (PAHs), acene dimers, pancake bond, non-covalent interactions (NCI), transition state (TS), DFT

## Abstract

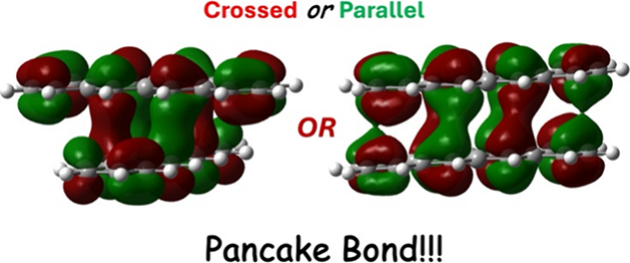

This study provides a systematic investigation of intermolecular interactions in homodimer of acenes using density functional theory (DFT). Focusing on the +1-charged dimers—frequently encountered in crystal structures—our analysis explores the influence of this charge, which introduces an unpaired electron, significantly affecting electronic properties. The interaction energy of +1-charged acene dimers is significantly larger compared to their neutral counterparts, attributed to the emergence of “pancake bonding″: a partially covalent interaction marked by intermolecular orbital overlap. This bonding mechanism contributes to the enhanced stability of charged acene dimers. Our findings indicate that the interplay between pancake bonding and van der Waals interactions influence the preferred orientations of monomers within these dimers. Transition state modeling reveals that orientational changes between dimer configurations do not completely break pancake bonds.

## Introduction

Intermolecular interactions between aromatic rings are crucial in chemistry and biology.^1,2^ Typically, these molecules are held together by van der Waals interactions, forming common π-stacking motifs. However, the presence of an unpaired electron in an organic ring with extended π-conjugation introduces a more intriguing scenario. The unique properties of organic radicals have garnered significant attention due to their potential applications in organic electronics.^3,4^ Despite their appeal, stabilizing organic radicals in the air remains a major challenge due to their high reactivity.^5,6^ Over the past decade, significant progress has been made in synthesizing open-shell organic materials,^7−10^ but a deeper understanding of the nature of their intermolecular interactions remains an active topic. Notable examples of the synthesis of neutral organic radicals with π-stacking in crystals are based on the phenalenyl motif, which has been provided by Nakasuji,^11^ Kubo,^12^ Haddon^13^ and others. Their stacking mechanism has been elucidated by Novoa,^14^ Kochi,^15,16^ Head-Gordon,^15^ Devic,^17^ and Kertesz, and their co-workers.^18−21^ One common approach to introducing an open-shell character into aromatic stacks is the synthesis of charge transfer salts of conjugated molecules with suitable counteranions. Significant research has focused on conducting charge-transfer salt TCNQ-TTF.^22−26^ Other charge transfer salts of aromatic radical cations such as naphthalene,^27,28^ anthracene,^29^ perylene,^30−32^ and triphenylene^33−36^ were also identified in the literature that form crystal phases with different counteranions. Although these counteranions are essential for stabilizing the crystal structure, they generally have minimal impact on π–π stacking interactions. These interactions are primarily governed by the electronic distribution within the conjugated molecules, independent of the nearby ionic charge. Charge transfer salts of conjugated molecules exhibit a wide range of π–π stacking motifs, including dimers, oligomers, and infinite stacks.^22,25,37^

Previous studies established the role of unpaired electrons in stabilizing π–π stacking interactions. It has been observed that when unpaired electrons are present within conjugated molecules, a unique type of bonding, known as a “pancake bond” (PCB), often forms.^38−40^ In the literature, pancake bonds are described as multicentered, n-electron (mc/ne) bonds, which result in a distinctive atom-overatom overlap in the π–π stacking arrangement, rather more commonly observed atom-over-ring overlap associated with van der Waals interactions.^17^ Pancake bond is partly covalent in nature,^14^ and its presence within the aromatic stacks makes the interplanar distance significantly shorter than the C···C van der Waals distance of 3.40 Å, and therefore, a more compact structure is typically observed. Many studies have demonstrated that mc/ne bonding develops when two antiaromatic rings align face-to-face.^41−46^ In such configurations, the bonding electrons are delocalized between the rings, substantially reducing the interplanar distance. Experimental evidence confirmed the presence of bonding electron density between the rings.^41^ while computational studies have shown an intermolecular orbital overlap between the π–electrons.^44^ Nevertheless, the interaction energy between the π–electrons in pancake-bonded stacks is significantly larger than the traditional π–π stacking motifs. Our recent investigations based on experiment and computation show the presence of PCB in perylene and triphenylene stacks at their charged states.^31,36,47^ For example, the contribution of pancake bond in +1-charged perylene dimers is computed to be 9.0 kcal/mol whereas for a triphenylene dimer, the same is computed as 11.9 kcal/mol. Generally, pancake bonds indicate highly delocalized electronic structures in the stacks which makes them attractive targets for designing highly conducting organic materials.

**Figure 1:**
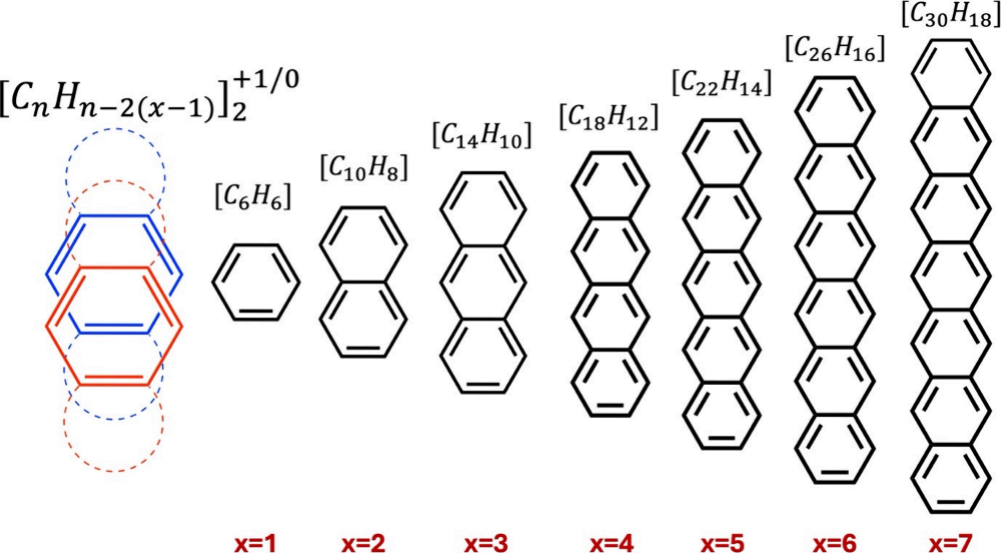
Schematical representation of the oligoacenes considers in this study. On the left is a symbolic representation of the oligoacene dimer with q = 0 or +1 additional dimer charge.

This study explores the nature of π–π stacking in a series of acene homodimers (benzene to heptacene, see Figure 1) assisted by pancake bonding when the total charge of the dimer is q = +1. For comparison, neutral (q = 0) dimers will also be discussed. These molecules constitute a series of polycyclic aromatic hydrocarbons (PAHs) that are under the right conditions prone to form π-stacking pancake bonded aggregates. The selection of the +1 charge is based on previous research on PAHs, which demonstrated that this charge state is highly conducive to the formation and strengthening of pancake bonds.^36,47,48^ While a + 2 charge can also promote pancake bonding, however, increasing the charge on the monomers tends to enhance electrostatic repulsion, weakening the interaction, and making it sometimes overall repulsive.^48^

In this report, a systematic investigation is provided on acene–acene interactions as the acene size increases from benzene to heptacene. Two types of dimer configurations are observed: crossed and parallel, as detailed later in the discussion. For shorter acene dimers, the crossed configuration is more stable, while for longer acenes, the parallel configuration becomes preferred. However, pancake bonding is present in all dimer types with a + 1 charge.

The study suggests that the interplay between the van der Waals interaction and pancake bond strength is the primary factor influencing the preference for different conformations. By investigating a series of PAHs we aim to obtain insights into the size dependency of π-stacking pancake bonding.

## Computational Methods

All calculations were performed using density functional theory (DFT) with the Gaussian 16 RevA.03 suite of programs.^49^ The M05-2X^50^ functional in combination with the 6-311G(d) basis set was employed for all computations, using the unrestricted (U) version for systems with unpaired electrons and the restricted open-shell (RO) formalism for generating molecular orbital diagrams. The choice of the former is based on a previous benchmark study by Mou et al.^51^ Structure optimizations were carried out with the default convergence criteria of Gaussian 16. Frequency calculations confirmed that all the optimized structures are ground-state minima without any imaginary modes. The interaction energy in the dimers is calculated using the eq 1 as defined below:
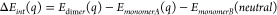
1

Transition state geometries were initially obtained using the QST2 tool and subsequently optimized using the “opt = ts″^49^ keyword to ensure a true transition state. The presence of a single imaginary vibrational mode validated the nature of the transition states. Furthermore, intrinsic reaction coordinate (IRC) calculations were performed to confirm connectivity between the transition states and their corresponding reactants and products.

To characterize the diradical/multiradical nature of the dimers in both neutral and cationic states, fractional occupation number-weighted electron density (N_FOD_) calculations were performed.^52^ The N_FOD_ values estimate the number of “hot” or unpaired electrons, as proposed by Grimme and Hansen. These calculations were performed using the ORCA 5.0.4 program^53^ at the B3LYP/def2-TZVP^54^ level of theory, with an electronic Fermi temperature set to T_e_ = 9000 K, as recommended.^55^ The N_FOD_ values correlate with the “number of effectively unpaired electrons” which were obtained using the high-level MR-AQCC method earlier.^56^

Unless otherwise specified, an isosurface value of 0.018 au is used for plotting orbitals, while a value of 0.0008 au is applied for the spin density plots.

## Results and Discussion

Introducing a + 1 charge is particularly intriguing, as it instills an unpaired electron within the dimer. This unpaired electron can delocalize across the dimer, potentially imparting unique electronic and intermolecular bonding properties to the dimer. To get deeper insights into such mechanism, we considered a series of acene dimers, generally formulated as [*C*_*n*_*H*_*n*–2(*x*–1)_]_2_^+*q*^(q = 0 or 1), where x ranges from 1 for the benzene (n = 6) dimer to 7 for the heptacene (n = 30) dimer; n is the number of carbon atoms in the acene.

**Figure 2:**
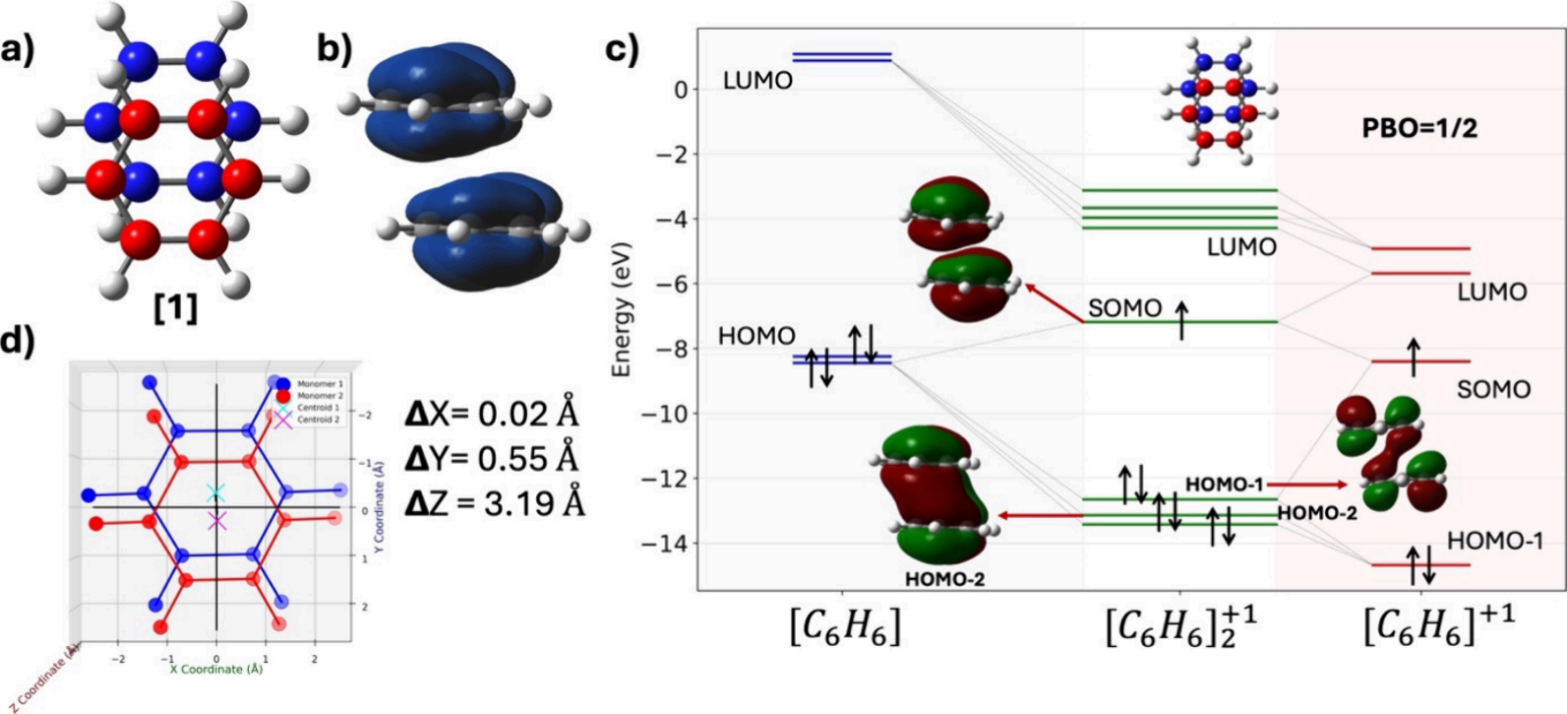
a) Optimized structure of the +1-charged benzene dimer, [*C*_6_*H*_6_]_2_^+1^ . b) Spin density of the benzene dimer. c) Molecular orbital (MO) diagram of [*C*_6_*H*_6_]_2_^+1^ considering four valence orbitals from the neutral and +1-charged benzene monomer. d) Relative positions of the benzene monomers in the +1-charged dimer.

The +1-charged benzene dimer([*C*_6_*H*_6_]_2_^+1^) is the smallest member of this acene series, making it an ideal representative system for detailed investigation. As seen in Figures 2a and 2d, the benzene monomers in the optimized [*C*_6_*H*_6_]_2_^+1^ structure are slipped parallel by 0.55 Å. The interaction energy between the benzene molecules in the dimer is calculated to be −20.5 kcal/mol. Notably, the enthalpy of interaction is computed as −18.9 kcal/mol, a value that aligns well with the previously reported experimental values of −17.0 kcal/mol by Meot-Ner^57^ in 1980 and later −17.6 kcal/mol by Rusyniak et al.^58^ For comparison, the interaction energy between two benzene molecules in the neutral dimer is calculated to be −2.8 kcal/mol.^59^ Thus, the −17.7 kcal/mol larger interaction energy in the +1-charged dimer relative to the neutral dimer confirms the presence of interactions beyond van der Waals, identified as pancake bonding in these systems.

To further characterize the pancake bond in [*C*_6_*H*_6_]_2_^+1^, a molecular orbital (MO) diagram has been generated considering the seven most relevant valence electrons, see Figure 2c. When neutral benzene interacts with a +1-charged benzene, one of the degenerate HOMOs of the neutral molecule couples strongly with the SOMO of the cation. As illustrated in Figure 2c, this coupling results in the formation of one doubly occupied bonding orbital (BO) which is (HOMO–2) orbital here, and a singly occupied antibonding molecular orbital (ABO), SOMO, primarily responsible for the formation of the pancake bond in the dimer. Note that the bonding vs antibonding characteristics in this case refer to the intermolecular interactions. Consequently, the formal bond order (FBO), or pancake bond order (PBO) between the monomer in the dimer is calculated as 1/2. The PBO is calculated using the equation below.
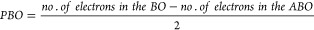
2

**Figure 3:**
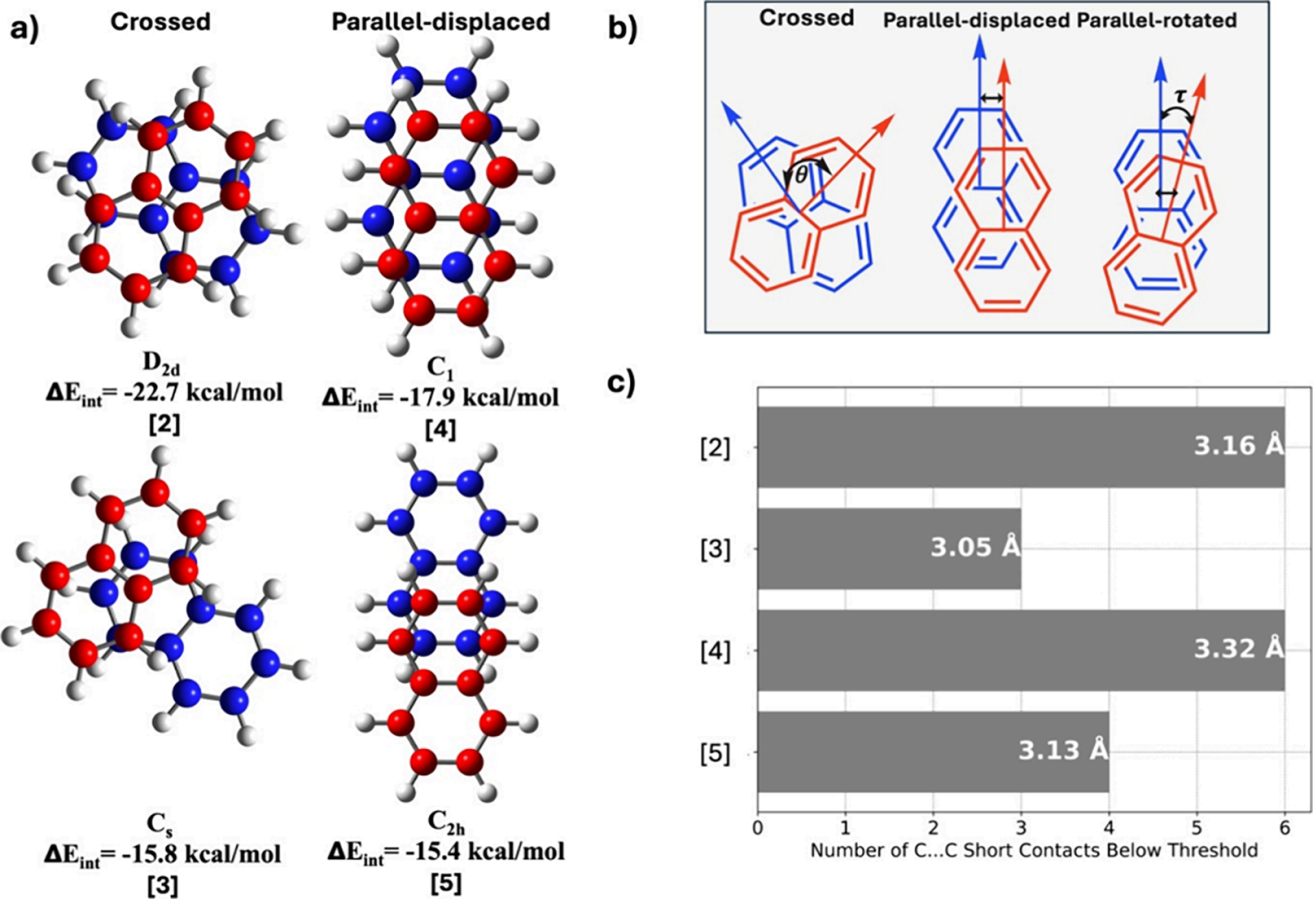
a) Optimized structures of the four conformers of ([*C*_10_*H*_8_]_2_^+1^), with the point groups and interaction energies listed below each structure. b) Schematic representation of the crossed, parallel-displaced, and parallel-rotated dimers. c) Bar chart showing the number of C···C short contacts in each dimer, where C···C contacts shorter than the selected threshold of 3.38 Å are considered. White numbers are average distances, as defined in eq 3.

The next member in the acene series is naphthalene. With two fused benzene rings, multiple structures of the naphthalene +1-charged dimer ([*C*_10_*H*_68_]_2_^+1^), were identified in the geometry optimizations. These dimers can be categorized as either crossed or parallel (displaced or rotated), depending on the relative orientation of the monomers, as shown in Figure 3a-b. Crossed dimers, depicted in Figure 3b, are characterized by nearly perpendicular alignment (θ ∼ 90°) of the acene chain lengths, while parallel dimers have their acene monomers aligned in the same direction or slightly rotated (τ ∼ 0°).

Figure 3a illustrates four distinct dimers—two of the crossed type and two of the parallel-displaced type. The interaction energy is highest in the crossed dimer [2], calculated at −22.7 kcal/mol. The other crossed dimer has a significantly lower interaction energy, which is expected due to reduced overlap between the monomers. Among the parallel-displaced dimers, [4] is the most stable, with an interaction energy of −17.9 kcal/mol. Figure 3c provides a comparative structural analysis of the four dimers. The gray bars represent the number of C···C short contacts, with the white numbers indicating the average C···C contact distances *d*_*av*_ computed according to eq 3.
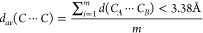
3

A distance of 3.38 Å, slightly shorter than the typical van der Waals distance of 3.40 Å for C···C interactions, was chosen to identify the short contacts with the total number of these contacts represented by *m*. Both structures [2] and [4] exhibit six C···C short contacts; however, the average distance between the naphthalene monomers is shorter in [2] at 3.16 Å compared to 3.32 Å in [4]. In dimers [3] and [5], although the interplanar distances are short, the lower number of short C···C contacts and less overlap between the monomers contribute to their smaller interaction energies.

**Figure 4:**
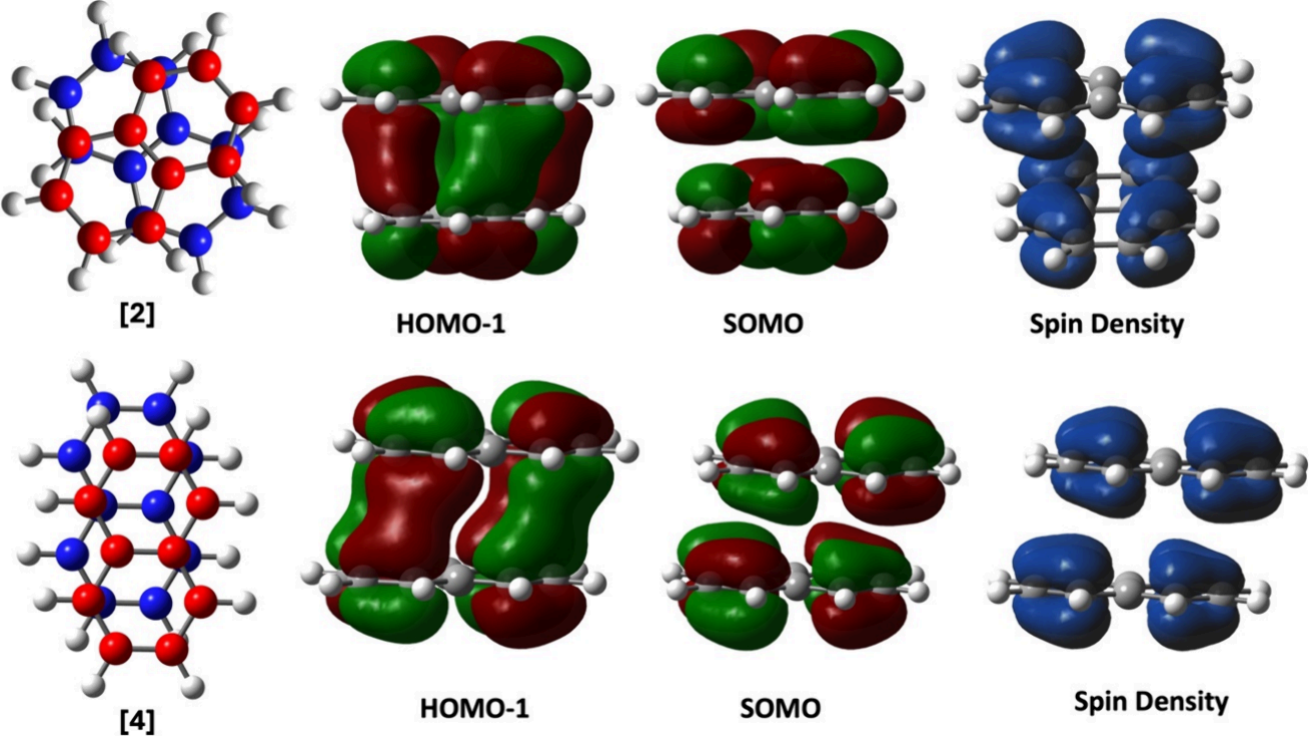
Properties of the two most stable dimer structures of [*C*_10_*H*_8_]_2_^+1^. The top row displays the HOMO–1 and SOMO, along with the spin density of conformer [2] of [*C*_10_*H*_8_]_2_^+1^. The bottom row presents the HOMO–1 and SOMO of conformer [4] of [*C*_10_*H*_8_]_2_^+1^.

The interaction energy between the two lowest energy conformers of [*C*_10_*H*_8_]_2_^+1^ [2] and [4] have been calculated at −22.7 kcal/mol and −17.9 kcal/mol, respectively. On the other hand, the interaction energy for the neutral naphthalene dimer is −5.6 kcal/mol at the presented level of theory, consistent with the previously reported value of about −5.7 kcal/mol by Tsuzuki et al.^60^ This indicates that in both dimers [2] and [4], the naphthalenes are bonded with much stronger interaction, with additional energies of −17.1 kcal/mol and −12.3 kcal/mol for [2] and [4], respectively. Like in the benzene dimer case, these enhanced interactions in the [*C*_10_*H*_8_]_2_^+1^ dimers are consistent with the presence of pancake bonds. The characteristic intermolecular orbital overlap observed in the HOMO–1 reassures the presence of PCB in these two +1-charged naphthalene dimers, as depicted in Figure 4. The antibonding nature of the SOMO calculates pancake bond order (PBO) of 1/2 in both cases. Additionally, the spin density plot shows an equal distribution of the unpaired electron across the dimer, corroborating equal charge sharing.

Although there are only a few examples, some crystal structures do exhibit the naphthalene-naphthalene cationic radical motif. Our CSD^61^ search identified three relevant crystal structures identified here by their CSD refcodes: DIPLAP11,^27^ JOSWET,^62^ and YETMUG.^28^ In all of these structures, the naphthalene units are functionalized with groups such as -OMe, -SMe or -Me, and no structure was found containing just naphthalene. The orientation of the monomers in the [*C*_10_*H*_8_]_2_^+1^ system is parallel-rotated in DIPLAP11,^27^ and JOSWET,^62^ while in YETMUG^28^ it adopts a crossed configuration.

**Figure 5:**
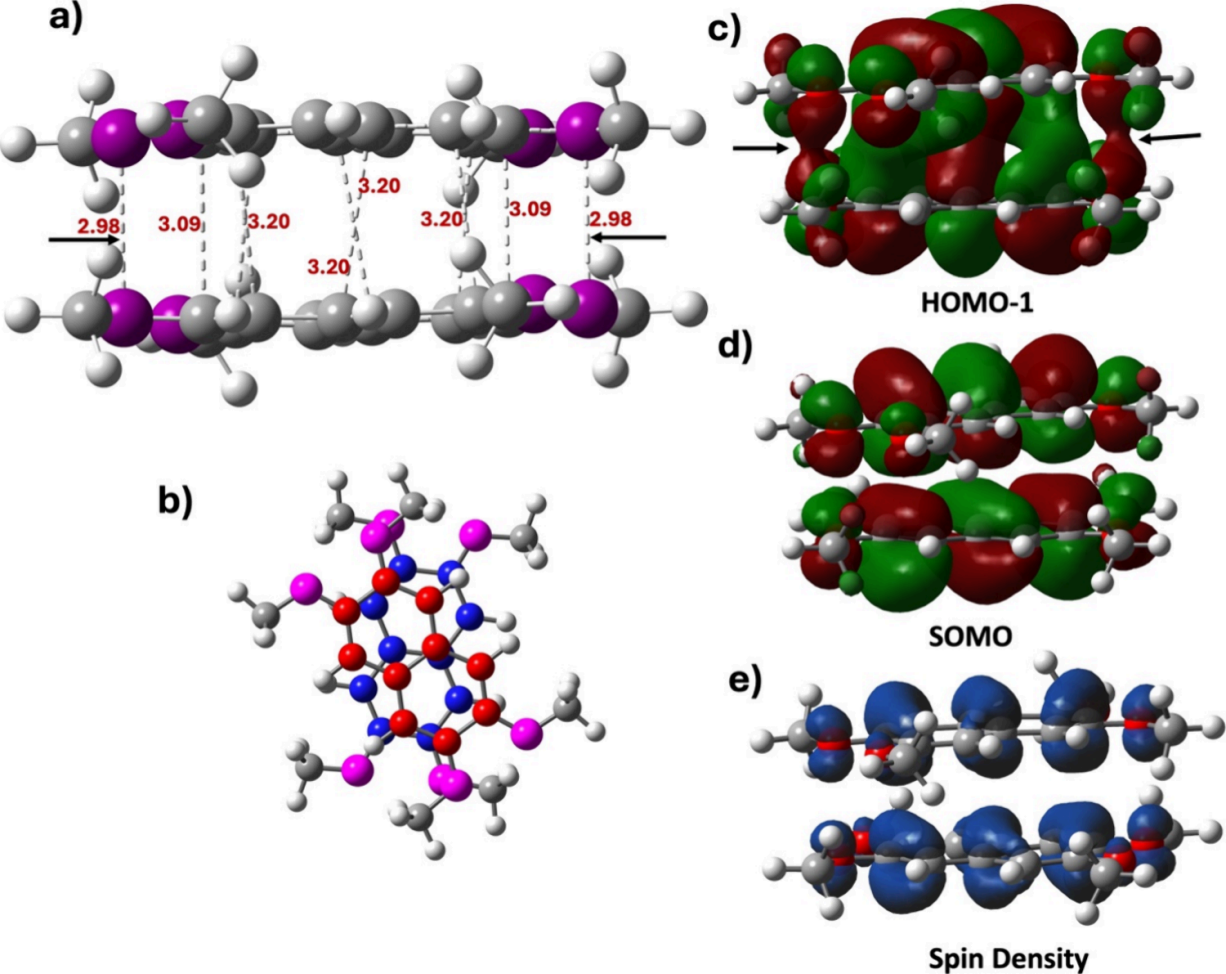
a) Optimized structure of the +1 charged cofacial dimer of 2,3,6,7-tetramethoxy naphthalene carved from the crystal structure with CSD entry code DIPLAP11.^27^ All short contacts are shown by dashed lines, the black arrows indicate the O···O short contacts b) Top view of the same dimer which shows the parallel-rotated configuration [color code aromatic carbon (top): red, aromatic carbon (below): blue, sp3 carbon: gray, oxygen: magenta, hydrogen: white]; c) HOMO–1 of the dimer; d) SOMO; e) spin density. An isosurface value of 0.012 au is used.

Interestingly, while -OMe and -SMe-substituted naphthalene dimers are stabilized in a parallel-rotated configuration in the ground state, substituting -OMe or -SMe with -H in the same motif leads to a crossed dimer as the optimized structure. To explore the factors contributing to the stability of the parallel-rotated arrangement in the -OMe and -SMe-substituted dimers, we optimized the dimer motifs excised from the crystal structures of DIPLAP11 and JOSWET. The results show that these dimers maintain the parallel-rotated configurations (with slight relaxation). The optimized structure of the dimer from DIPLAP11, illustrated in Figure 5a, contains eight short C···C contacts, with distances ranging from 3.09 to3.20 Å.

What is most intriguing is the presence of two short O···O contacts at 2.98 Å each, as shown in Figure 5a. This value is to be compared with the O···O van der Waals contact distance of 3.04 Å.^63^ To further investigate the nature of this O···O intermolecular interaction, we analyzed the HOMO–1 and SOMO, which are found to be pivotal in the pancake bond formation as discussed above. Alongside the C···C overlaps, the HOMO–1 in Figure 5c clearly reveals significant intermolecular O···O orbital overlap, which is crucial for locking the structure into the parallel-rotated configuration. The pancake bond order remains 1/2 in this dimer as the SOMO is antibonding to the HOMO–1 and the spin density is equally distributed in the dimer, shown in Figure 5e.

The interaction energy between two 2,3,6,7-tetramethoxy naphthalene units is calculated to be −30.6 kcal/mol, 6 kcal/mol more stable than that of the bare naphthalene dimer, as discussed earlier. Additionally, the interaction energy for the neutral 2,3,6,7-tetramethoxy naphthalene dimer was calculated to be −19.1 kcal/mol, estimating the contribution of the pancake bond in the cationic dimer to be approximately 11.5 kcal/mol. Based on this analysis, it can be argued that the stabilization of the methoxy-substituted naphthalene dimer in the parallel-rotated form arises from the additional O···O intermolecular overlap. In the absence of this interaction, the parallel-rotated form becomes unstable, leading the structure to relax into an alternative ground state minimum. Similar observations were made from the optimized dimer from the JOSWET crystal structure, and the results are illustrated in Figure S1 in the Supporting Information. Two S···S short contacts at 3.41 Å (<S···S vdW distance is 3.60 Å^63^) along with orbital overlap are identified to be the key to stabilizing the dimer in parallel-rotated form.

**Figure 6:**
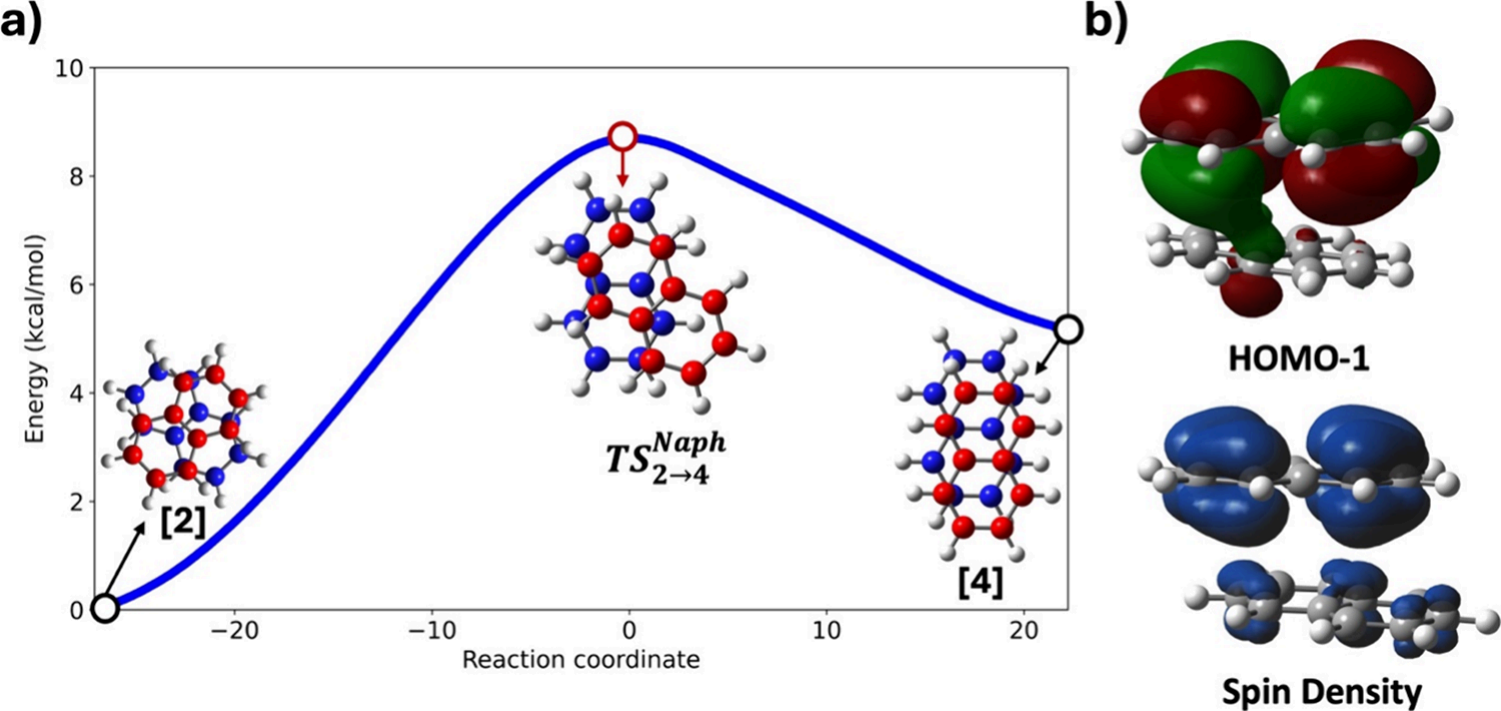
a) Potential energy surface (PES) for the conversion of the crossed +1-charged naphthalene dimer [2] to the parallel-displaced +1-charged naphthalene dimer [4] via the transition state **TS**_2 → 4_^Naph^. b) Uneven HOMO–1 orbital and total spin density of the transition state **TS**_2 → 4_^Naph^.

Exploring the transformation pathway from the crossed dimer to the parallel-displaced dimer is further intriguing. Since both dimers involve pancake bonds, sliding one monomer over the other is not straightforward and requires the rupture and reformation of the pancake bond. Figure 6a illustrates the reaction coordinate for the transformation from [2] to [4]. This process passes through a transition state, **TS**_**2 → 4**_^**Naph**^, with an activation energy (Δ*E*^‡^) of 8.8 kcal/mol relative to [2]. As previously noted, the interaction energy between naphthalene in [2] is −22.7 kcal/mol, and after subtracting the van der Waals contribution, the pancake bonding interaction is −17.2 kcal/mol. Thus, if the pancake bond were completely lost in **TS**_**2 → 4**_^**Naph**^, we would expect an activation barrier of around 17 kcal/mol, which is not observed here. The lower activation energy suggests that the pancake bond is still partially retained in the transition state, as discussed below.

A closer examination of the transition state reveals five short C···C contacts, measuring 3.12 Å, 3.17 Å, 3.22 Å, 3.30 Å, and 3.33 Å. However, the HOMO–1 shows only one significant intermolecular orbital overlap, which contributes to the stabilization of the transition state and lowers the activation barrier. Additionally, the spin density of **TS**_**2 → 4**_^**Naph**^ displays an unequal distribution, consistent with the observed unequal charge distribution. CHelpG charge analysis shows that in **TS**_**2 → 4**_^**Naph**^, one naphthalene unit carries a charge of +0.78, while the other carries +0.22. This unequal electron distribution in the transition state weakens the pancake bonding compared to the two respective minima.

A remarkable feature of this PES is that there is only a single TS present, somewhat similar as seen in the case of perylene cation dimer.^47^ Along the reaction coordinate the multicenter pancake bond does not completely break, even though the displacements are significant. It appears that sufficient electron sharing remains present as the two molecules slide over one another and maintain a measure of pancake bonding even at geometries that are not fully overlapping (see Figure S2). This observation has broader consequences in assessing intermolecular bonding interactions driven by pancake bonding.

We now turn to the larger members of the series introduced in Figure 1 paying particular attention to the various stable conformations of the cation dimers. In the case of the anthracene cation dimer, [C_14_H_10_]_2_^+^, we have identified four distinct dimer structures ([6], [7], [8], [9]), as depicted in Figure S3 dimer [6] is classified as a crossed dimer, while the remaining three are categorized as parallel-displaced, based on the earlier definitions. The interaction energy for the crossed dimer [6] is calculated to be −21.8 kcal/mol which is the most stable of the four. The parallel-displaced dimer with the maximum overlap [7], has an interaction energy of −19.7 kcal/mol, which is the most stable among the three parallel-displaced structures Though unsubstituted anthracene favors the crossed-dimer form, a methyl-substituted anthracene dimer with a + 1 charge in a parallel-displaced configuration is identified in the CSD database.^61^ The crystal structure with CSD refocde QETGEB^29^ contains a + 1 charge octamethyl anthracene dimer in the unit cell, stabilized by an SbCl_6_^–^ counterion. This high degree of substitution may favor the parallel arrangement. Nevertheless, these results indicate that both crossed and parallel-displaced dimers are more stable when the overlap between monomers is maximized. Structural analysis reveals that dimers [6], [7], and [8] exhibit six short C···C contacts, while dimer [9] has four, as shown in Figure S2. Among these, dimer [6] has the shortest average C···C distance (d_av_), measuring 3.17 Å. To confirm the presence of a pancake bond in the anthracene dimer, we plotted the HOMO–1 of dimers [6] and [7], clearly illustrating large intermolecular overlap. Additionally, the SOMO of both [6] and [7] exhibits intermolecular antibonding characteristics, confirming PBO = 1/2 for both dimers. Key orbitals and spin density of [6] and [7] are provided in Figure S4.

**Figure 7:**
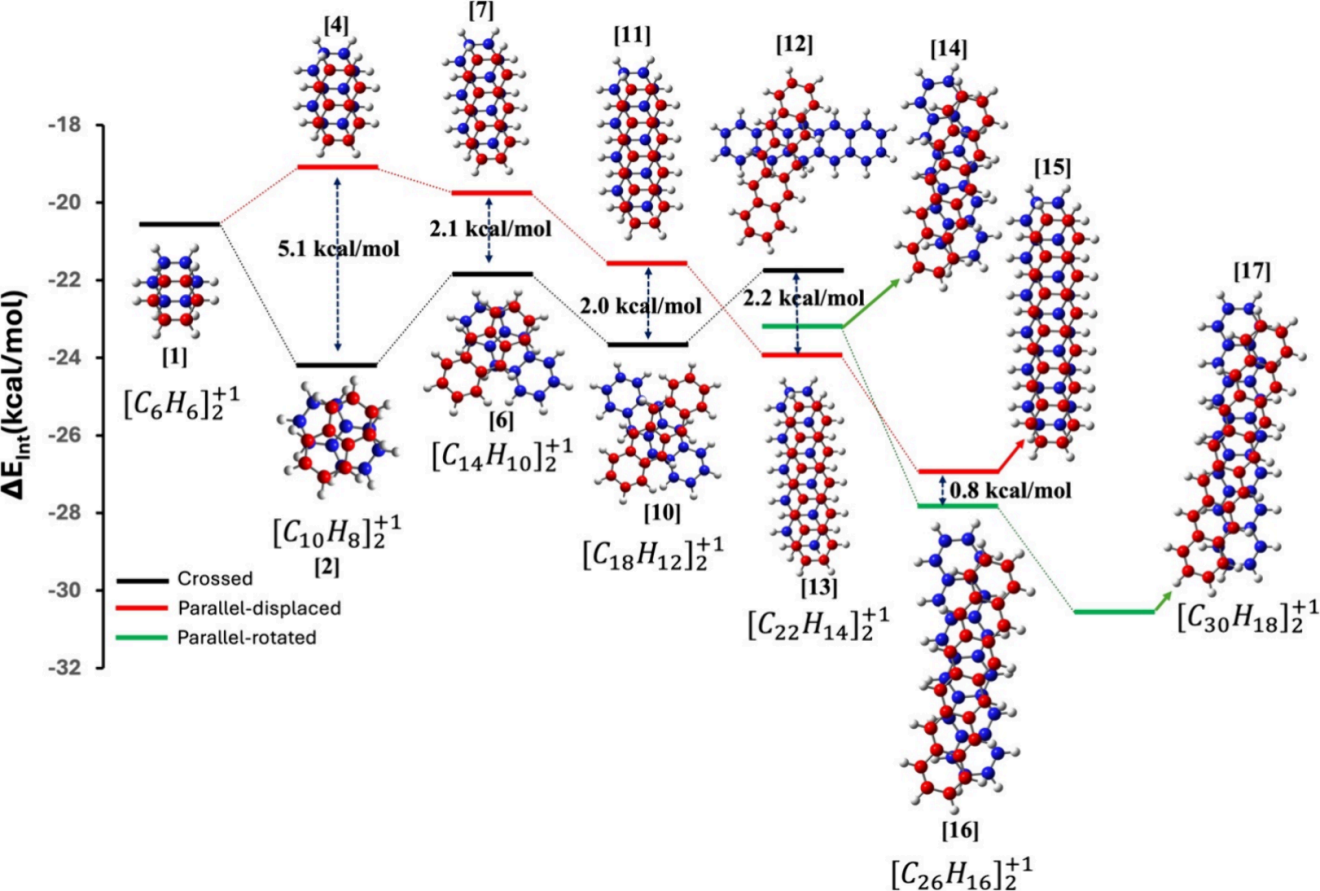
Interaction energy diagram of +1-charged acene dimers from benzene (1-acene) to heptacene (7-acene). For benzene, only one stable conformer is possible, while for hexacene and heptacene, no crossed dimer was found.

Calculations on [C_10_H_8_]_2_^+^ and [C_14_H_10_]_2_^+^ indicate that both the crossed and parallel-displaced configurations are more stable when the overlap between monomers is significant. Accordingly, for larger acene systems such as tetracene, pentacene, hexacene, and heptacene dimers, we have focused on optimizing the crossed dimers and the parallel-displaced dimers with maximum overlap.^17^Figure 7 presents the interaction energies of the most stable crossed and parallel dimers, along with their relative stabilities.

In the case of benzene, only one dimer configuration is possible. As previously discussed, the crossed dimer is more stable than the parallel-displaced form in both naphthalene and anthracene dimers. However, the energy gap between these two configurations decreases by 3.0 kcal/mol for the anthracene dimer. This trend continues with tetracene, where the crossed dimer is 2.0 kcal/mol more stable than the parallel-displaced one (see also Figure S5). Interestingly, with pentacene dimers, the situation shifts. In addition to the crossed and parallel dimer forms, we also identified a new configuration resembling the parallel form but with a slight rotation, which we refer to as the *parallel-rotated* dimer and shown in Figure 7 with a green line (see also Figure S6). In the case of pentacene, unlike the previous systems, the parallel dimers are the most stable. Specifically, the parallel-displaced dimer is the most stable, being 2.2 kcal/mol lower in energy than the crossed dimer, while the dimer is only 0.8 kcal/mol higher in energy than the parallel-displaced form. Moving on to the hexacene dimer, we were able to optimize only the parallel and the parallel-rotated dimer configurations, as all attempts to stabilize a crossed dimer failed. For hexacene, the parallel-rotated dimer is actually the most stable, with an energy of 0.8 kcal/mol lower than the parallel-displaced analog. Finally, with heptacene, we observed only one type of dimer, the parallel-rotated form, as attempts to optimize any other dimer configuration were unsuccessful.

The crossed dimer for tetracene shows a slight deviation from its ideal geometry, with this distortion becoming even more significant for the pentacene dimer. For hexacene and heptacene, a stable crossed dimer configuration could not be obtained, as all optimization attempts instead converged to an almost parallel arrangement. The increased stability of the pentacene and longer acene dimers in the parallel configuration is likely due to their extended chain length, which promotes greater overlap and stronger van der Waals interactions, outweighing the higher pancake bond strength in the crossed dimers.

**Figure 8:**
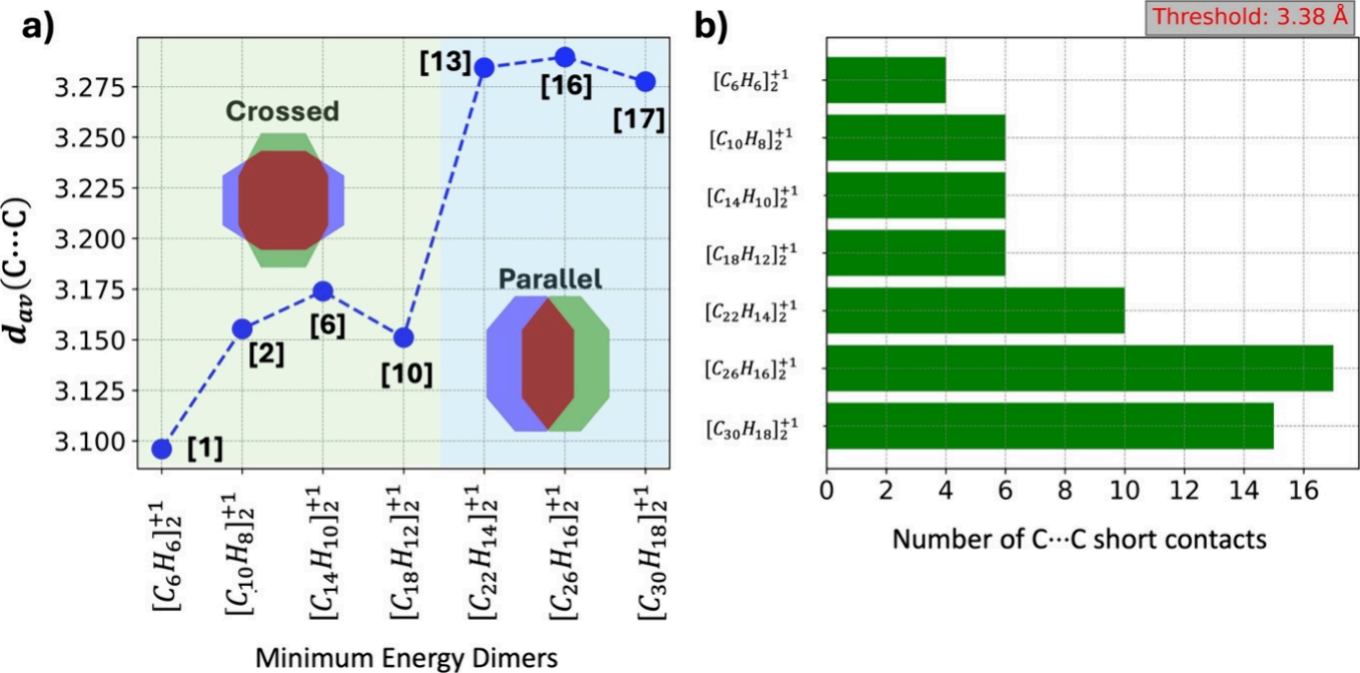
a) Average C···C contact distances in minimum-energy +1-charged acene dimers, calculated using eq 3. The first four data points, shown in the green-shaded region, represent crossed dimers, while the last three represent parallel or parallel-rotated dimers as obtained from the minimum-energy configurations are shown in Figure 7. b) Number of C···C short contacts in the +1-charged dimers, with distances below a threshold of 3.38 Å.

First, we discuss the trends in the geometries of the most stable cation dimers. Figure 8a illustrates the average C···C short contacts (d_av_(C···C)) within the +1-charged acene dimers from benzene to heptacene. The first four points, representing dimers from benzene to tetracene, exhibit a crossed configuration as the most stable form and are shown within a green-shaded zone. The remaining three dimers, which favor a parallel or parallel rotated configuration as the most stable minima, are highlighted in a blue-shaded zone. As seen in Figure 8a, there is a noticeable increase in the average C···C short contacts when the dimer configuration shifts from crossed to parallel. For the crossed dimers, d_av_ ranges between 3.10 and 3.18 Å, while for the parallel dimers, d_av_ spans from 3.27 to 3.28 Å. Figure 8b further reveals that the number of C···C short contacts (<3.38 Å) is significantly higher in the parallel dimers. This greater number of short contacts compensates for the reduction in pancake interaction due to the increased interplanar distances. Additionally, the larger number of C···C short contacts is consistent with the greater overlap observed in parallel dimers compared to crossed dimers.

**Figure 9:**
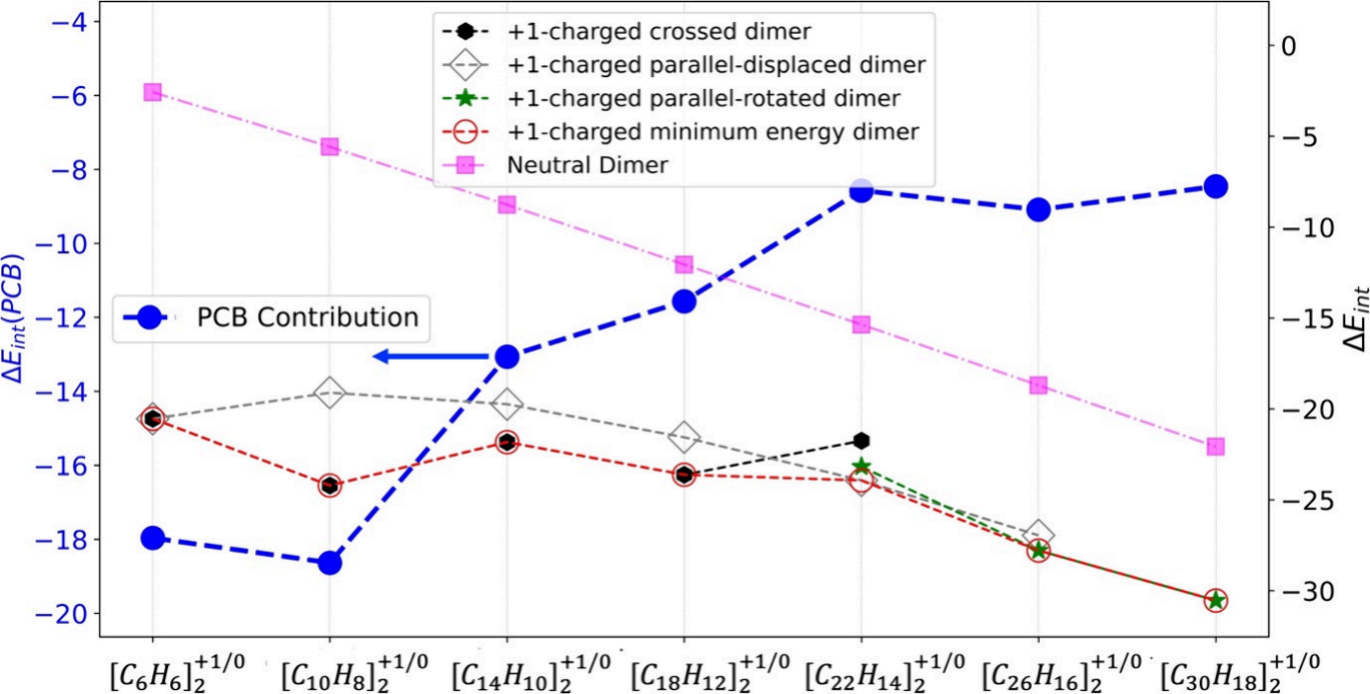
Interaction energy between monomers in the acene homodimer, [*C*_*n*_*H*_*n*–2(*x*–1)_]_2_^+1^. x varies from 1 to 7, corresponding to acenes from benzene (x = 1) to heptacene (x = 7). The black, gray, and green markers depict the interaction energies for the crossed, parallel-displaced, and parallel-rotated dimers, respectively. The red circles represent the low-energy +1-charged dimers, regardless of whether they are crossed, parallel-displaced, or parallel-rotated. The magenta squares show the interaction energy of the neutral dimers. The blue dots estimate the pancake contribution in the interaction energy (Δ*E*_int_(PCB)) in the +1-charged dimer and it is calculated by using the equation: Δ*E*_int_(PCB) = Δ*E*_int_(+1 charged minimum energy dimer) - Δ*E*_int_(neutral dimer).

The interaction energy within the dimer is key to identifying the presence of a pancake bond. Specifically, when the interaction energy in the +1-charged dimer is significantly greater than that of the corresponding neutral dimer, it suggests that an additional attractive interaction beyond van der Waals interactions is at play. In these dimers, the pancake interaction substantially stabilizes the charged dimer. As listed in Table 1, the contribution of this pancake bond (PCB) in the charged dimer can be approximated by the difference in interaction energy between the +1-charged dimer and the corresponding neutral dimer, denoted as Δ*E*_int_(PCB). These values are represented by the blue dots in Figure 9. It is important to note that the red circles represent the minimum energy of the +1-charged acene dimer: the first four points correspond to the crossed dimer, the fifth point corresponds to the minimum pentacene dimer which is parallel-displaced type, and the last two points are the energies for parallel-rotated dimers which are found to be minimum for hexacene and heptacene (cf. Figure 7). The gray and orange markers show the interaction energies of the low-energy crossed and parallel-displaced dimers, respectively. Meanwhile, the magenta line indicates the interaction energy between the acenes in their neutral states.

**Table 1:** Interaction Energies, in kcal/mol, between the Acene Monomers in the Neutral and +1-Charged Dimers^a^

Minimum Energy Dimer	Δ*E*_int_(q = +1)	Δ*E*_int_(q = 0)	Δ*E*_int_(PCB)	Configuration
[C_6_H_6_]_2_^q^	–20.5	–2.6	–17.9	-
[C_10_H_8_]_2_^q^	–24.2	–5.6	–18.6	crossed
[C_14_H_10_]_2_^q^	–21.8	–8.8	–13.1	crossed
[C_1__8_H_1__2_]_2_^q^	–23.6	–12.0	–11.6	crossed
[C_22_H_1__4_]_2_^q^	–23.9	–15.4	–8.6	parallel-displaced
[C_26_H_1__6_]_2_^q^	–27.8	–18.7	–9.1	parallel-rotated
[C_30_H_1__8_]_2_^q^	–30.5	–22.1	–8.5	parallel-rotated

a Only the most stable dimer is listed for each. From the difference in the interaction energies in the +1-charged and neutral dimer, the contribution of the pancake bond in the dimer formation is estimated. Δ*E*_int_(PCB) = Δ*E*_int_(q = +1) – Δ*E*_int_(q = 0) is the estimated pancake bond contribution.

Interestingly, the interaction energy in the +1-charged dimer remains relatively constant from naphthalene to pentacene, as shown by the red circles in Figure 9. There is a slight decrease in the benzene dimer and a slight increase in the hexacene dimer. In contrast, the interaction energy for the neutral dimers increases linearly with acene size. This linear dependency on size is a reflection of the extensive nature of van der Waals interactions. By implication, the increasing diradical character of the neutral oligoacenes^64^ seems to play no role in affecting this trend significantly.

So why does not the interaction energy follow the same trend in the charged dimers? The answer lies in the blue line in Figure 9. As the acene size increases, there is greater overall surface overlap (not orbital overlap) between the monomers, leading to stronger van der Waals interactions. However, the pancake interaction within the dimers weakens as the acene size grows. For the benzene and naphthalene dimers, Δ*E*_int_(PCB) is highest, but it decreases linearly in absolute value until the pentacene dimer. After pentacene, Δ*E*_int_(PCB) reaches a plateau, with the values remaining nearly constant for pentacene, hexacene, and heptacene dimers. As the length of the acene unit increases, the strength of the pancake bond converges to a limiting value of ∼8 kcal/mol for the dimers. At this size, the vdW interaction is larger than the pancake bond contribution. Intermolecular packing at this point is not dominated for the larger members of the series by the maximum overlap.

**Figure 10:**
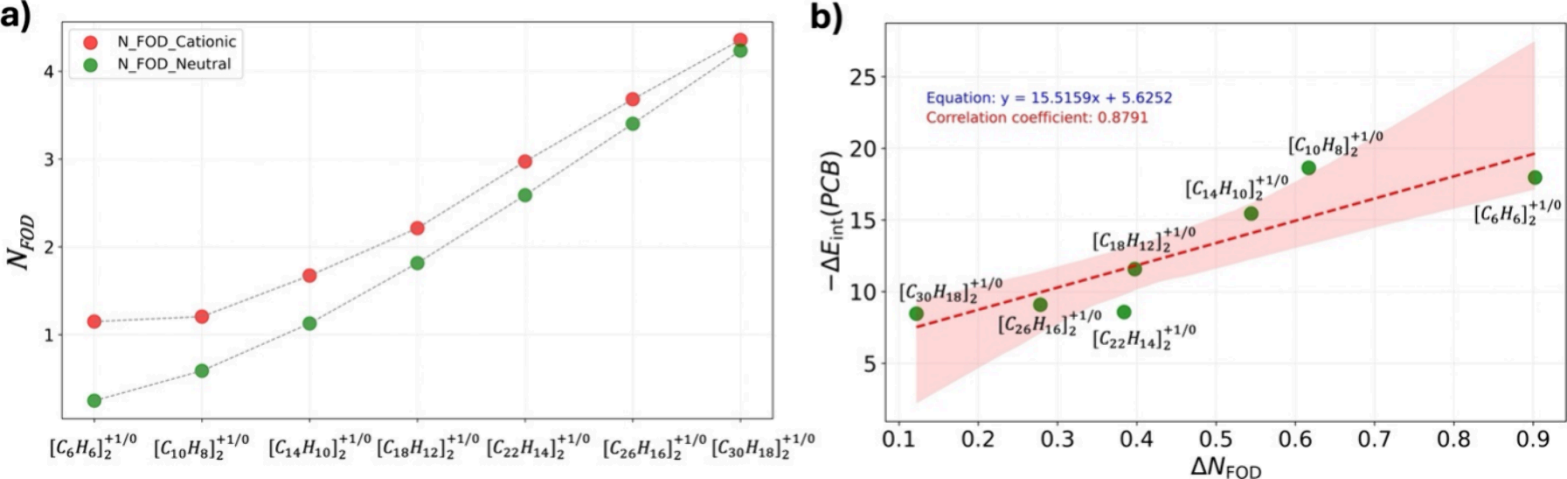
a) N_FOD_ values for all the acene dimers (+1 charged in red and neutral in green) considered in this study. b) Correlation between the ΔN_FOD_ values and the pancake bond contribution in the interaction energy (Δ*E*_int_(PCB)) for cationic acene dimers. Here, ΔN_FOD_ = N_FOD_(cationic dimer) - N_FOD_(neutral dimer).

An interesting insight into the intermolecular interaction in the oligoacene dimers series is obtained by comparing their open vs closed shell characters.^64^ In order to estimate the number of unpaired or “hot” electrons within these systems, we calculated the N_FOD_ values for both +1-charged and neutral acene dimers, focusing on minimum energy structures (shown as red circles in Figure 9) and presenting these values in Figure 10a. N_FOD_, a modern and cost-efficient descriptor of the polyradical index in a system, was introduced by Grimme and Hansen and has proven reliable for aromatic molecules.^65^ Note that as with any descriptor of the polyradical character, N_FOD_ is not a physical observable and should be used only for comparisons. To validate its accuracy within the presented level of computational theory, we have compared the N_FOD_ values for the neutral acene chains from naphthalene (2-acene) to decacene (10-acene) to the number of effectively unpaired electrons (N_u_) obtained using a high level of theory at the MR- AQCC level from a previous report by Plasser et al.^56^ As provided in the Supporting Information in Figure S7, the correlation between the DFT-predicted N_FOD_ values and the MR-AQCC-predicted N_u_ values are excellent with a correlation coefficient of 0.98. The latter reassures our choice of the N_FOD_ index for the current investigation. The N_FOD_ values for cationic acene dimers are indicated by the red dots in Figure 10a, while the green dots denote the corresponding values for the neutral dimers. As anticipated, the N_FOD_ values for the cationic dimers are larger than those of the neutral dimers, reflecting a greater radicaloid nature. However, the trend between cationic and neutral dimers begins to converge with increasing acene length, with the N_FOD_ values almost merging in the case of the heptacene dimer.

In another attempt, we sought to analyze correlation between the differences between N_FOD_ (cationic and neutral) and the PCB contribution to interaction energy. Figure 10b shows a good correlation (coefficient = 0.88), suggesting a relationship between N_FOD_ differences and PCB contributions for the acene dimers. As the difference between the N_FOD_ values of +1-charged and neutral acene dimer decreases step-by-step, the Δ*E*_int_(PCB) values also decrease significantly. Based on this analysis, it is hypothesized that for larger cationic dimers, the monomers are more effective at stabilizing these hot electrons due to increased chain length, thereby reducing the number of electrons available for PCB formation. Consequently, this stabilization effect may result in a diminished PCB contribution in larger acene dimers.

Although the focus of this article is not on heterodimers, we briefly explore the implications when two different acene monomers interact with a total charge of +1. Specifically, we examine the heterodimer formed by benzene and naphthalene for two reasons: (i) this pairing creates the smallest possible heterodimer, allowing for straightforward interpretation, and (ii) a detailed analysis of this system has been previously reported by Attah et al.^66^

**Figure 11:**
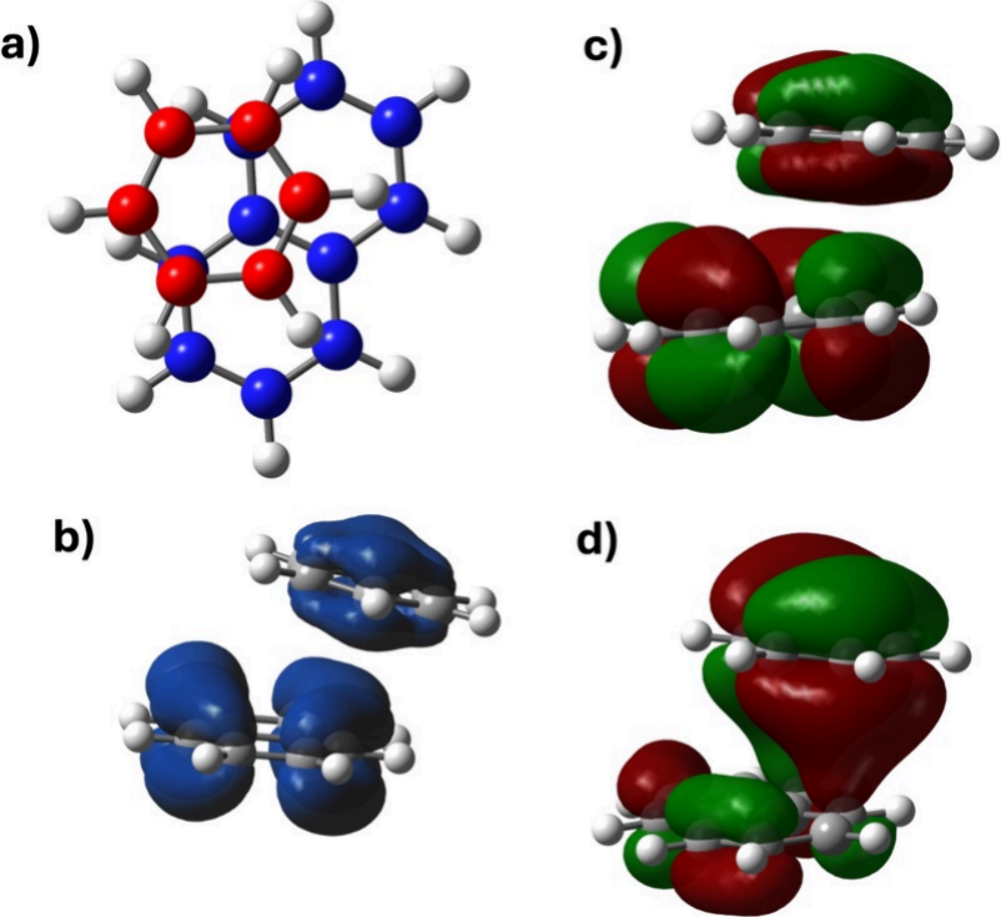
(a) Top view of the optimized structure, (b) spin density, (c) highest occupied molecular orbital (HOMO), and (d) lowest unoccupied molecular orbital (LUMO) of the of the [(C_10_H_8_)(C_6_H_6_)]^+^ heterodimer.

Figure 11a illustrates the optimized structure of a benzene molecule positioned above a naphthalene molecule, representing the global minimum for the [(C_10_H_8_)(C_6_H_6_)]^+^ system.^66^ The interaction energy between benzene and naphthalene in this heterodimer is calculated to be −10.2 kcal/mol using the same DFT as in the rest of this article, which aligns well with the experimental value of −7.9 ± 1 kcal/mol and the computed value of −8.4 kcal/mol.^66^ Notably, this interaction energy is significantly weaker than that of the benzene homodimer [(C_6_H_6_)_2_]^+^ and the naphthalene homodimer [(C_10_H_8_)_2_]^+^. We attribute this reduced interaction energy to the weaker pancake bonding due to low charge delocalization in the heterodimer, as indicated by the lower orbital overlap in the HOMO–2 (Figure 11d).

The +1 charge in [(C_10_H_8_)(C_6_H_6_)]^+^ is unevenly distributed, with naphthalene carrying a larger share (+0.79) compared to benzene (+0.21), as calculated from the CHelpG charge analysis. This unequal charge distribution is further corroborated by the spin density distribution shown in Figure 11b. In [(C_1_0H_8_)(C_6_H_6_)]^+^, only 21% of the charge is delocalized, resulting in a pancake bond strength (Δ*E*_int_(PCB)) of −8.5 kcal/mol, which is significantly lower than the corresponding values for the benzene homodimer (−17.9 kcal/mol) and the naphthalene homodimer (−18.6 kcal/mol).

## Conclusions

This study presents the analysis of the interactions between acene monomers within their respective homodimers in their π-stacking configurations. A range of acene homodimers, from benzene to heptacene, were investigated, with the total charge, q, of the dimers constrained to either neutral (0) or +1. Across all cases, the intermolecular interactions in the +1-charged dimers were significantly stronger than in their neutral counterparts. Particularly, shorter acene dimers showed a significantly greater enhancement of interaction in the q = +1 state compared to their neutral analogs; a trend less pronounced in longer acenes. This increased interaction in the +1-charged dimers is attributed to the formation of a pancake bond between the monomers, which was confirmed by identifying the specific orbitals involved. The bond order for these pancake interactions in all +1-charged dimers was determined to be 1/2.

By studying the transition among two π-stacking local minima of the [*C*_10_*H*_8_]_2_^+1^ naphthalene cation dimer, one crossed, the other parallel slipped, we discovered that the less than ideally overlapping configurations still maintain a certain amount of pancake bonding. This result can explain why pancake-like bonding is found in a number of crystal structures where the partial loss of pancake bonding is likely compensated by crystal packing and cation–anion electrostatics.

The relative orientation of the monomers in the dimers is dependent on acene size. Smaller acene dimers tend to adopt a crossed π-stacking configuration, while larger ones are found to be more stable in a π-stacking parallel arrangement. However, in substituted dimers, the preferred orientation can differ. In the cases of the -OMe and -SMe substituted naphthalene dimers, the O···O and S···S orbital overlaps were identified as the key factors stabilizing the experimentally observed π-stacking parallel-rotated form of the +1-charged dimers.

The open-shell character of the wave function as monitored by the N_FOD_ index is shown to play a significant role in the presented intermolecular interactions. The variation in N_FOD_ values between neutral and +1-charged dimers correlates with the contribution of the pancake bond to the overall interaction energy.

Close packing of cationic PAH π-stacking dimers is a structural motif that promotes pancake bonding and high intermolecular electronic overlap. It appears that the size dependency of this effect in oligoacenes prefers relatively small and intermediate sizes in the series. At this point we cannot assess the generality of this observation, but the implications for crystal design are present.

In heterodimers, the contribution of pancake bonding to the interaction energy is expected to be lower due to uneven charge delocalization, which reduces orbital overlap. The extent of this uneven charge distribution depends on the difference in ionization potentials between the constituent rings and the shapes of the relevant orbitals.

## Supporting Information Available

Images of HOMOs and LUMOs, number of short contacts plot, spin densities, structural comparison (PDF)
Caretesian coordinatess (XYZ)
Video of sliding motion of naphthalenes in a +1-charged homodimer during interconformer conversion (MP4)

## Supplementary Material

mg4c00153_si_001.pdf

mg4c00153_si_002.xyz
