# Supplementary material for: Pancake Bonding in the Stabilization of Cationic Acene Dimers
Source: ACS Mater Au. 2025 Jan 30;5(2):365–76. doi: 10.1021/acsmaterialsau.4c00153 (PMC11907286; doi:10.1021/acsmaterialsau.4c00153)
Supplement: Supplementary file 1 — mg4c00153_si_001.pdf [file mg4c00153_si_001.pdf]

Supporting Information For

Pancake Bonding in the Stabilization of Cationic Acene Dimers

Rameswar Bhattacharjee[1]*, Hans Lischka[2], Miklos Kertesz[1]*

[1] Chemistry Department and Institute of Soft Matter, Georgetown University
37th and O Streets, NW, Washington, DC 20057-1227, USA
[2] Department of Chemistry and Biochemistry, Texas Tech University, Lubbock, TX 79409, USA

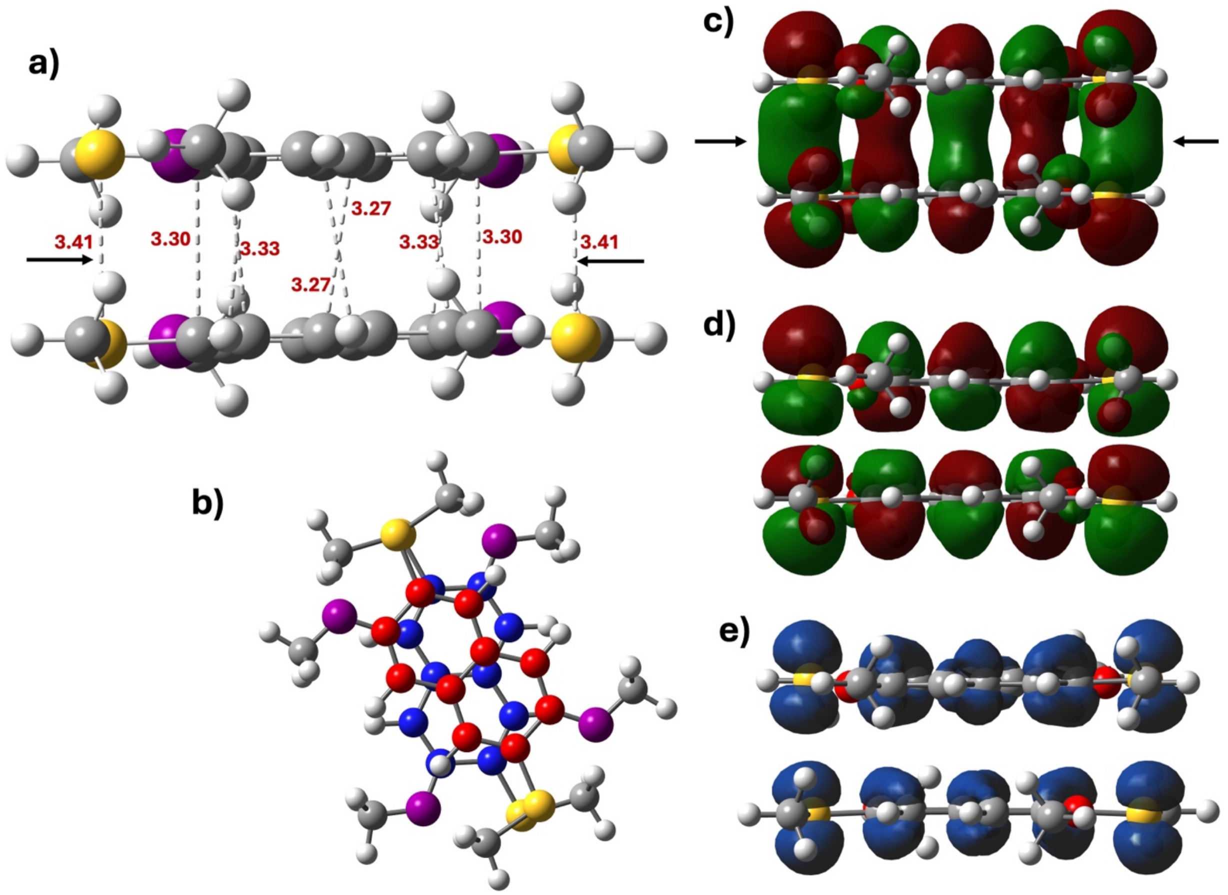

Figure S1: a) Optimized structure of the +1 charged cofacial dimer of [2, 6-dimethoxy-3, 7-bis (methylthio) naphthalene] carved from the crystal structure with CSD entry code JOSWET[1] . All short contacts are shown by dashed lines. b) Top view of the same dimer which shows the parallel-rotated configuration [color code aromatic carbon(top): red, aromatic carbon (below) : blue, sp3 carbon: grey, oxygen: magenta, sulfur: yellow hydrogen: white]; c) HOMO-1 of the dimer, the black arrows indicate the S···S short contacts ; d) SOMO; e) spin density. An isosurface value of 0.012 a.u. is used.

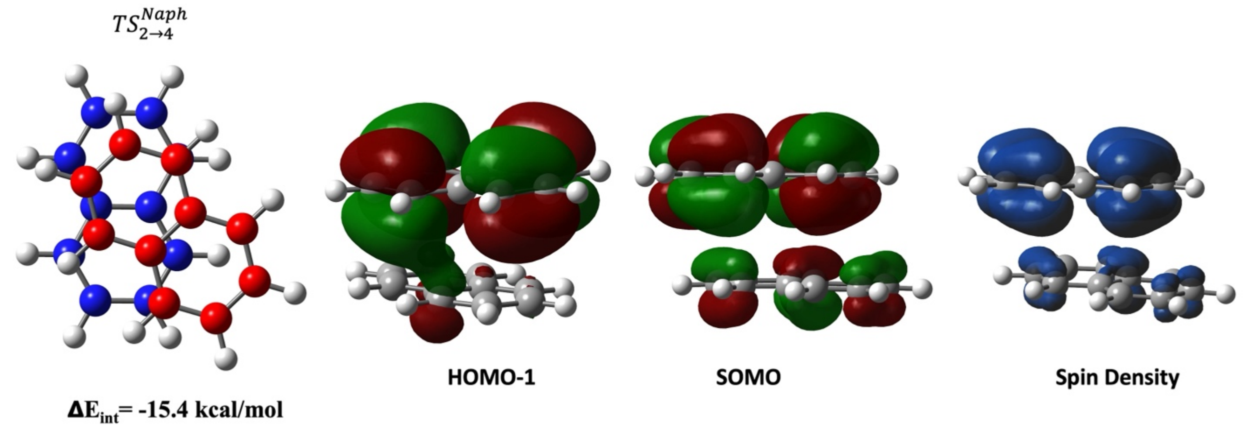

Figure S2: Optimized structure of the transition state, $\boldsymbol{TS^{Naph}_{2\to4}}$, the HOMO-1 and HOMO, and the spin density.

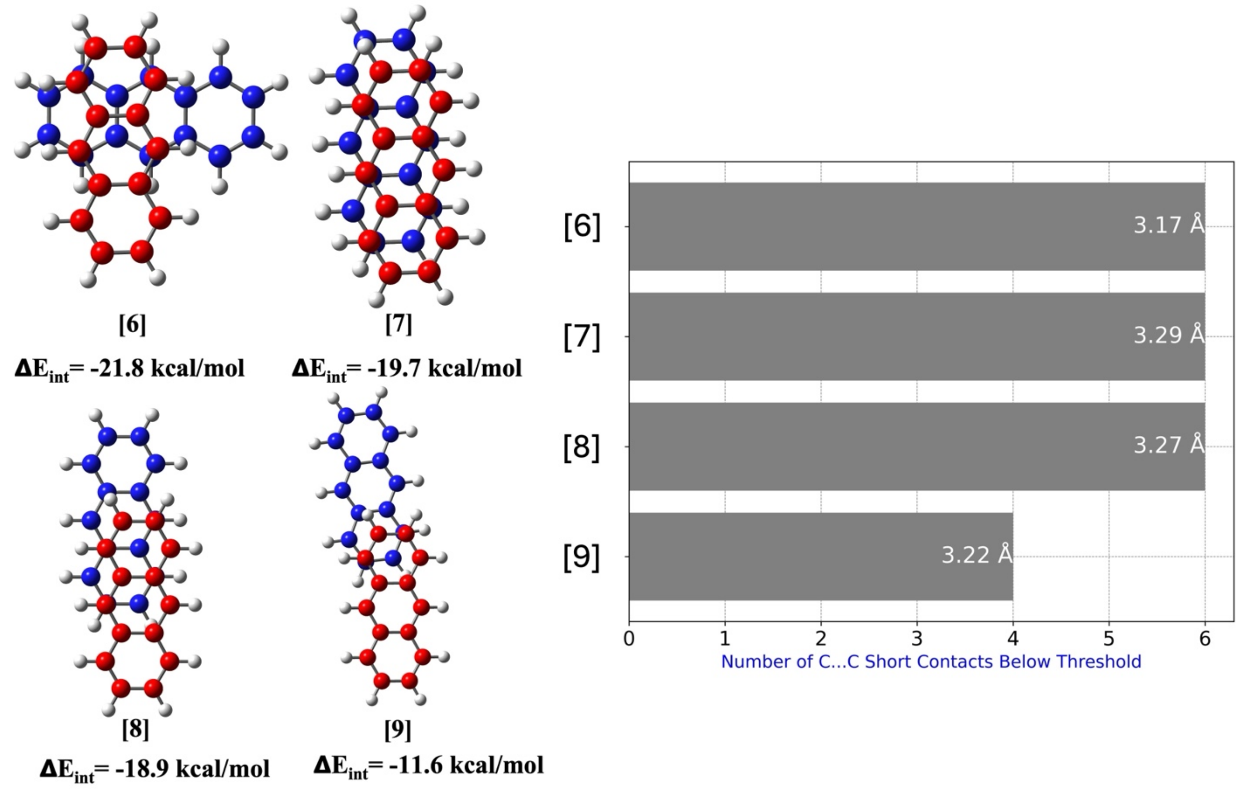

Figure S3: Optimized structure of four different configurations of +1-charged anthracene dimer, $[C_{14}H_{10}]_2^+$, with the interaction energy between the anthracenes in dimer listed below each of them. The bar plot on the right side shows the number of C···C short contacts and the d_{av} value for each of the dimer structure. A threshold value of 3.38 Å is used to identify the short contacts.

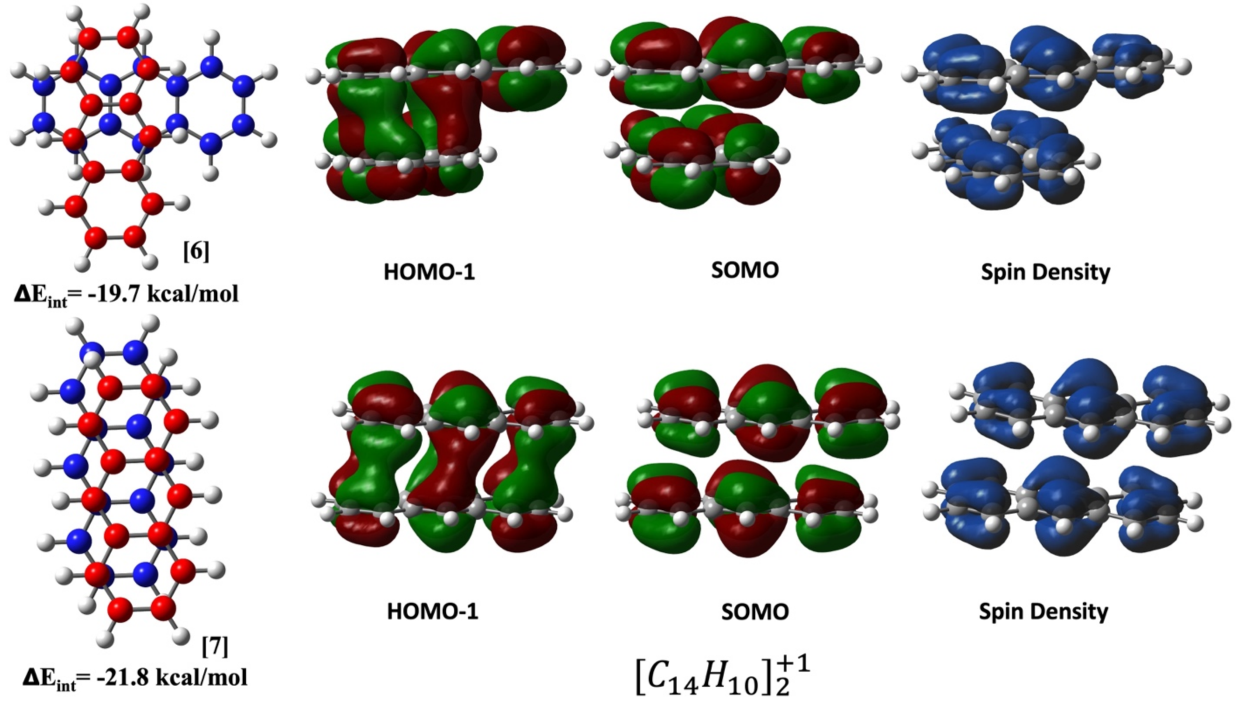

Figure S4: key orbitals, HOMO-1, SOMO of two relevant anthracene dimers. The spin densities are also shown.

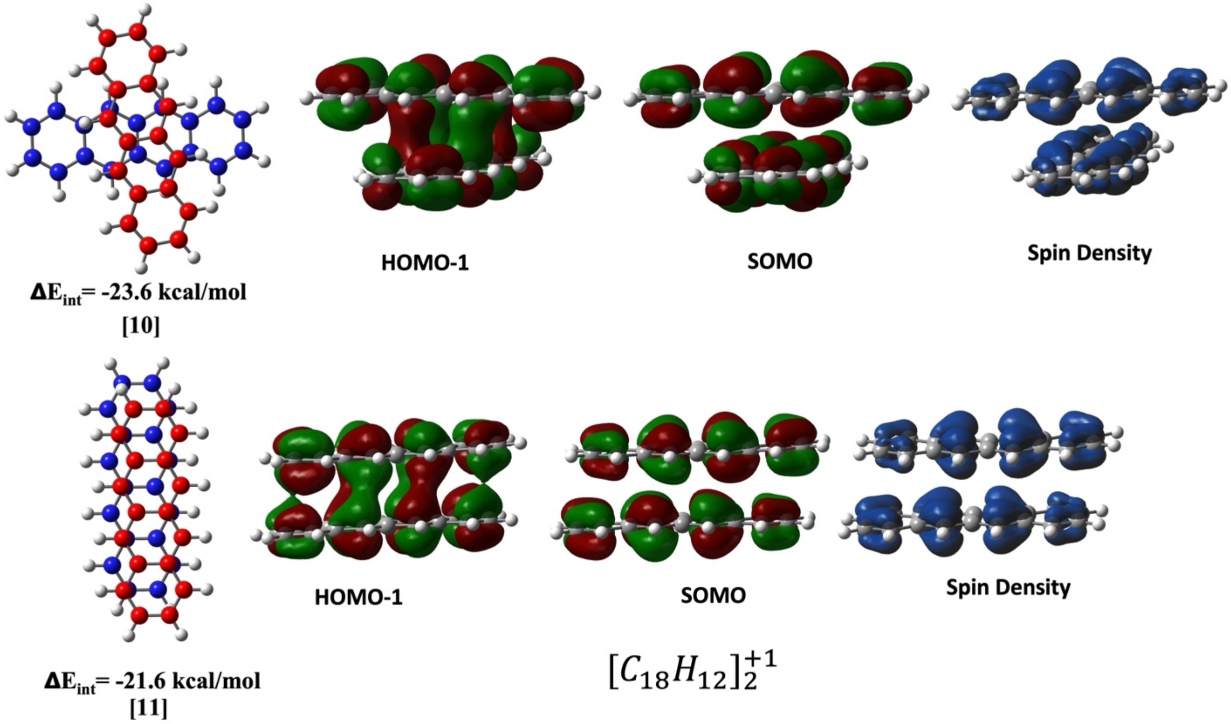

Figure S5: Key orbitals, HOMO-1, SOMO, of two relevant tetracene dimers. The spin densities are also shown.

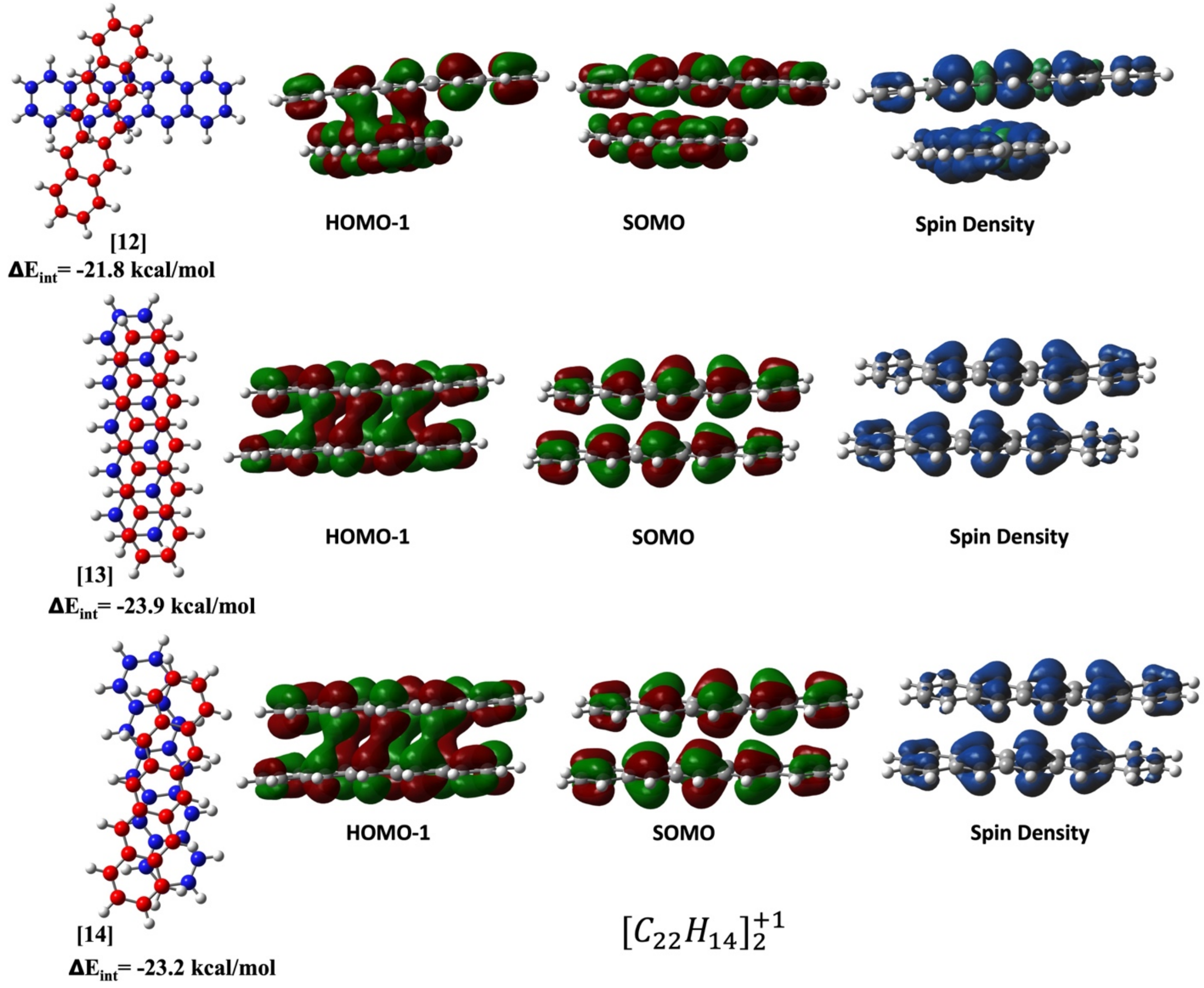

Figure S6: Key orbitals, HOMO-1, SOMO, of two relevant pentacene dimers. The spin densities are also shown.

Table S1: The angle between two acene chains in the three relevant q=+1 charged acene dimers discussed in the manuscript.

Structure Index	τ
[14]	24.3°
[16]	20.9°
[17]	18.2°

Table S2: Key structural parameters of the dimer, derived from the crystal structure with CSD entry DIPLAP11,[2] before and after optimization.

CSD Entry: DIPLAP11	Expt.[2]	Optimized
τ	34.0°	44.6°
O···O 1	3.35 Å	2.98 Å
C···C 1	3.29 Å	3.09 Å
C···C 2	3.33 Å	3.21 Å
C···C 3	3.31 Å	3.21 Å
C···C 4	3.29 Å	3.20 Å
C···C 5	3.29 Å	3.20 Å
C···C 6	3.24 Å	3.09 Å
C···C 7	3.33 Å	3.21 Å
C···C 8	3.38 Å	3.21 Å
O···O 9	3.22 Å	2.98 Å

Table S3: Key structural parameters of the dimer, derived from the crystal structure with CSD entry JOSWET[1], before and after optimization.

CSD Entry: JOSWET	Expt.[1]	Optimized
τ	43.4°	44.6°
S···S 1	3.40 Å	3.41 Å
C···C 1	3.42 Å	3.30 Å
C···C 2	3.46 Å	3.33 Å
C···C 3	3.47 Å	3.33 Å

C···C 4	3.41 Å	3.27 Å
C···C 5	3.41 Å	3.27 Å
C···C 6	3.42 Å	3.30 Å
C···C 7	3.47 Å	3.33 Å
C···C 8	3.46 Å	3.33 Å
S···S 2	3.40 Å	3.41 Å

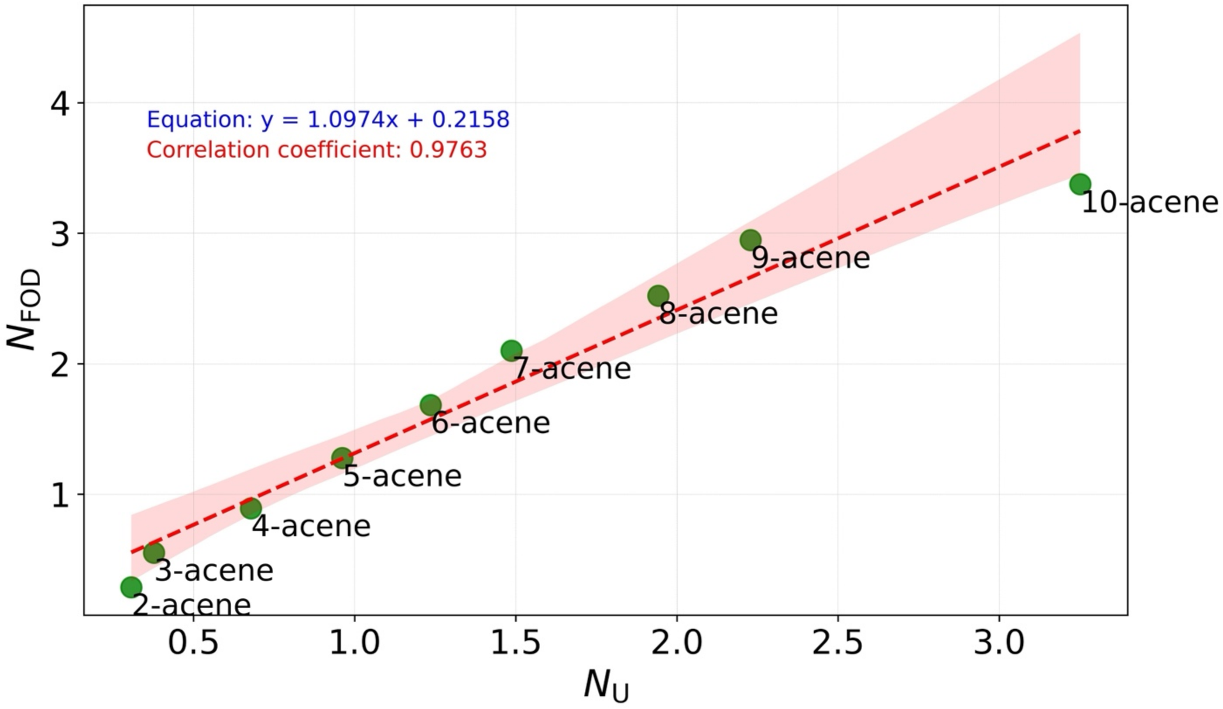

Figure S7: Correlation between the N_{FOD} obtained from DFT values from M05-2X/6-311G(d) and N_u values obtained from MR-AQCC for neutral acene monomers.[3] In Ref ([3]) a slightly different basis set was used with an almost identical correlation as shown here.

References:

(1) Noreland, J.; Olovsson, G.; Olovsson, I. Structure of the organic semiconducting radical cation salt tris [2, 6-dimethoxy-3, 7-bis (methylthio) naphthalene] diperchlorate. *Acta Crystallogr.,Sect.C:Cryst.Struct.Commun.* **1992**, *48* (8), 1459-1462.

(2) Olovsson, G.; Olovsson, I. Structure of the organic semiconducting radical cation salt tris (2, 3, 6, 7-tetramethoxynaphthalene) bis (hexafluoroarsenate). *Acta Crystallographica Section B: Structural Science* **1991**, *47* (3), 355-362.

(3) Nieman, R.; Carvalho, J. R.; Jayee, B.; Hansen, A.; Aquino, A. J.; Kertesz, M.; Lischka, H. Polyradical character assessment using multireference calculations and comparison with density-functional derived fractional occupation number weighted density analysis. *Phys. Chem. Chem. Phys.* **2023**, *25* (40), 27380-27393.
